# Renal Safety of [^177^Lu]Lu-PSMA-617 Radioligand Therapy in Patients with Compromised Baseline Kidney Function

**DOI:** 10.3390/cancers13123095

**Published:** 2021-06-21

**Authors:** Florian Rosar, Niklas Kochems, Mark Bartholomä, Stephan Maus, Tobias Stemler, Johannes Linxweiler, Fadi Khreish, Samer Ezziddin

**Affiliations:** 1 Department of Nuclear Medicine, Saarland University, 66421 Homburg, Germany; florian.rosar@uks.eu (F.R.); s8nikoch@stud.uni-saarland.de (N.K.); mark.bartholomae@uks.eu (M.B.); stephan.maus@uks.eu (S.M.); tobias.stemler@uks.eu (T.S.); fadi.khreish@uks.eu (F.K.); 2 Department of Urology, Saarland University, 66421 Homburg, Germany; johannes.linxweiler@uks.eu

**Keywords:** PSMA radioligand therapy, ^177^Lu, safety, kidney, renal function, metastatic castration-resistant prostate carcinoma

## Abstract

**Simple Summary:**

Radioligand therapy (RLT) targeting prostate-specific membrane antigen (PSMA) is an effective antitumor-treatment in metastatic castration-resistant prostate carcinoma (mCRPC). Concerns of potential nephrotoxicity are based on renal tubular PSMA expression and the resulting radiopharmaceutical retention during RLT, but data confirming clinically significant renal toxicity are still lacking. In this study, *n* = 22 patients treated within a prospective patient registry (REALITY Study) with significantly impaired baseline kidney function were investigated for treatment-associated nephrotoxicity and the potential relationship with administered activities of [^177^Lu]Lu-PSMA-617. As opposed to prevailing concerns, glomerular filtration rate (GFR) improved significantly in our cohort of patients and no significant correlation between change in GFR and administered activities were found. As pre-treatment chronic kidney failure did not lead to detectable RLT-induced deterioration of renal function in our study, the nephrotoxic potential of [^177^Lu]Lu-PSMA-617 RLT may be overestimated. We suggest not to categorically exclude patients from enrolment to PSMA-RLT due to renal impairment.

**Abstract:**

*Background:* Radioligand therapy (RLT) targeting prostate-specific membrane antigen (PSMA) is an effective antitumor-treatment in metastatic castration-resistant prostate carcinoma (mCRPC). Concerns of potential nephrotoxicity are based on renal tubular PSMA expression and the resulting radiopharmaceutical retention during RLT, but data confirming clinically significant renal toxicity are still lacking. In this study, patients with significantly impaired baseline kidney function before initiation of therapy were investigated for treatment-associated nephrotoxicity and the potential relationship with administered activities of [^177^Lu]Lu-PSMA-617. *Methods:* Twenty-two mCRPC patients with impaired renal function (glomerular filtration rate (GFR) ≤ 60 mL/min) who received more than two cycles of [^177^Lu]Lu-PSMA-617 RLT (median 5 cycles and median 6-week time interval between consecutive cycles) were analyzed in this study. Patients were treated within a prospective patient registry (REALITY Study, NCT04833517). Cumulative administered activities ranged from 17.1 to 85.6 GBq with a median activity of 6.5 GBq per cycle. Renal function was closely monitored during and after PSMA-RLT. *Results:* Mean pre-treatment GFR was 45.0 ± 10.7 mL/min. After two (22/22 patients), four (20/22 patients), and six cycles (10/22 patients) of RLT, a significant increase of GFR was noted (each *p* < 0.05). End-of-treatment GFR (54.1 ± 16.7 mL/min) was significantly higher than baseline GFR (*p* = 0.016). Only one patient experienced deterioration of renal function (change of CTCAE grade 2 to 3). The remaining patients showed no significant reduction of GFR, including follow-up assessments (6, 9, and 12 months), and even showed improved (10/22 patients) or unchanged (11/22 patients) CTCAE-based renal impairment grades during and after the end of PSMA-RLT. No significant correlation between the change in GFR and per-cycle (*p* = 0.605) or cumulative (*p* = 0.132) administered activities were found. *Conclusions:* As pre-treatment chronic kidney failure did not lead to detectable RLT-induced deterioration of renal function in our study, the nephrotoxic potential of [^177^Lu]Lu-PSMA-617 RLT may be overestimated and not of clinical priority in the setting of palliative treatment in mCRPC. We suggest not to categorically exclude patients from enrolment to PSMA-RLT due to renal impairment.

## 1. Introduction

Besides established chemotherapy with taxanes (docetaxel, cabazitaxel) [1,2,3] and treatment with novel androgen axis drugs (NAAD) (abiraterone or enzalutamide) [4,5], radioligand therapy targeting the prostate-specific membrane antigen (PSMA) is a promising therapy option in metastatic castration-resistant prostate carcinoma (mCRPC) [6,7]. PSMA is a transmembrane glycoprotein, which is largely overexpressed on the cell surface of prostate cancer cells [8]. Its overexpression and endocytosis after radioligand binding to the protein makes PSMA a specific target offering new ways of imaging and treatment of prostate carcinoma [9,10]. PSMA-targeted radioligand therapy (PSMA-RLT) with [^177^Lu]Lu-PSMA-617 has shown promising results in various recent studies [11,12,13,14,15]. Adverse effects seem to be rare and related to irradiation of non-target tissue due to the physiologic PSMA expression in healthy organs, such as the salivary glands and the kidneys. One observed adverse effect is xerostomia negatively affecting the quality of life without being life threatening. Physiological PSMA expression in the cells of the proximal renal tubules could potentially lead to more serious complications. However, despite the high tracer accumulation in the kidneys, little has been reported on the renal toxicity of PSMA-RLT, which could be explained by the fact that most patients receive PSMA-RLT as a last-option treatment, and therefore, long follow-up data are rare. Nevertheless, higher grades of nephrotoxicity, i.e., grade ≥ 3, defined by common terminology criteria for adverse effects (CTCAE), seem not to occur in patients with normal renal function [16]. Yet, there is no study investigating nephrotoxicity in patients with renal impairment before commencement of PSMA-RLT, although renal impairment is not uncommon in advanced mCRPC settings and can result from nephrotoxic effects of previous androgen deprivation therapy (ADT) or taxane-based chemotherapy [17,18]. In addition, obstruction of the urinary tract by excessive iliac and retroperitoneal tumor masses may also lead to obstructive nephropathy with reduced renal function [19].

In this study, we investigated treatment-associated nephrotoxicity and its potential relationship with administered activities of [^177^Lu]Lu-PSMA-617 in mCRPC patients with impaired renal function at baseline, i.e., before commencement of PSMA-RLT.

## 2. Methods

In this study, *n* = 22 consecutive mCRPC patients with markedly impaired renal function, who received more than two cycles of [^177^Lu]Lu-PSMA-617 RLT in the palliative setting were analyzed. Patients were treated at our institution within a prospective patient registry (REALITY Study, NCT04833517). Impaired renal function was defined as a glomerular filtration rate (GFR) ≤ 60 mL/min [20]. GFR was estimated using the MDRD formula [21]. All patients had high tumor burden and had received various conventional treatments prior to PSMA-RLT, including ADT, NAAD, and chemotherapy. Table 1 presents the detailed patient characteristics.

PSMA-RLT was performed on a compassionate use basis under the German Pharmaceutical Act §13 (2b). All patients gave their written consent after being thoroughly informed about the risks and side effects of this new therapy and consented to publication of their data in accordance with the declaration of Helsinki. The study was approved by the local Institutional Review Board (ethics committee permission number 140/17).

The radioligand [^177^Lu]Lu-PSMA-617 was administered during an inpatient stay in accordance with German radiation protection regulations. Each patient received intravenous hydration (500 mL 0.9% NaCl) and cooling of the salivary glands, starting 30 min prior to treatment infusion. The [^177^Lu]Lu-PSMA-617 solution was intravenously administered by infusion line over a period of 1 h. No diuretics (except in patients with heart failure-related fluid restrictions) or other renal protection were applied. The patients were treated by PSMA-RLT with a median of five cycles (range: 3–14) and with a median time interval of 6 weeks between consecutive cycles. The administered activity ranged from 3.1 to 10.9 GBq per cycles (median and mean 6.5 GBq). The total administered activity of [^177^Lu]Lu-PSMA-617 ranged from 17.1 to 85.6 GBq (median 36.2 GBq, mean 40.1 GBq). Detailed information about the individual PSMA-RLT course of each patient is presented in the Supplementary Material (Figure S1). Renal function was closely monitored during and after PSMA-RLT. The median follow-up time was 10 months (range 3–24 month). For statistical analysis, Wilcoxon matched-pairs signed rank test, Spearman correlation, and Fisher’s exact test were applied using Prism 8 (GraphPad Software, San Diego, CA, USA). A *p*-value < 0.05 was regarded as statistically significant.

## 3. Results

**Figure 1:**
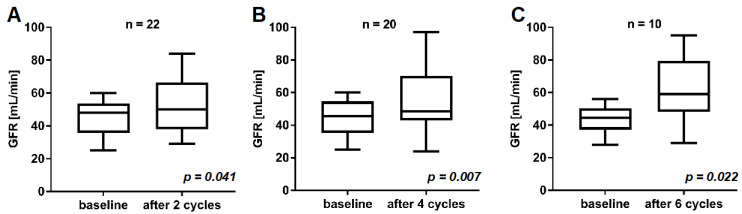
Comparison of GFR at baseline and after two cycles (**A**), four cycles (**B**), and six cycles (**C**) of [^177^Lu]Lu-PSMA-617 RLT.

**Figure 2:**
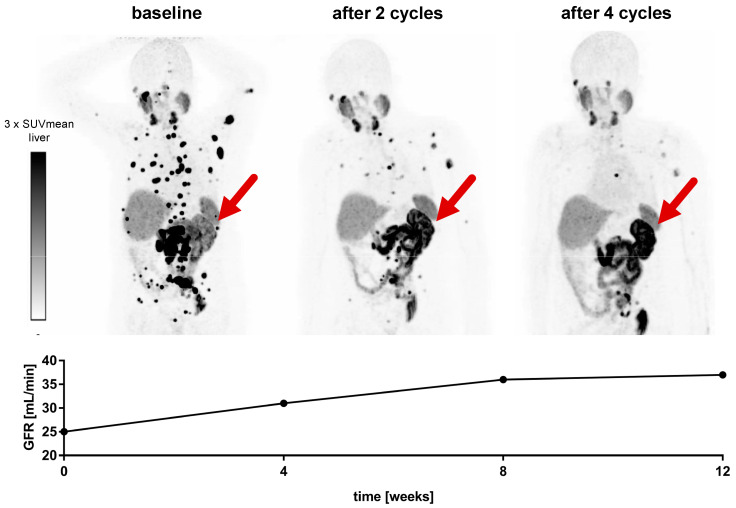
Advanced mCRPC patient with reduced condition and impaired renal function before commencement of PSMA-RLT. The patient received four cycles of [^177^Lu]Lu-PSMA-617. While marked tumor regression and improvement of the patient’s condition was observed, GFR increased from 25 mL/min at baseline to 37 mL/min after the end of [^177^Lu]Lu-PSMA-617 RLT. This improvement of renal function can also be seen on [^68^Ga]Ga-PSMA-11 PET imaging with an increased tracer uptake in the single left kidney (red arrow). The patient had a history of right kidney resection due to renal cell carcinoma.

In the cohort of *n* = 22 mCRPC patients included in this study, the mean baseline GFR before commencement of PSMA-RLT was 45.0 ± 10.7 mL/min. After two, four, and six cycles of [^177^Lu]Lu-PSMA-617 RLT, no decrease, but a significant increase of renal function was observed (Figure 1). After two cycles, GFR was 52.0 ± 17.4 mL/min (vs. 45.0 ± 10.7 mL/min at baseline, *p* = 0.041). In total, 20/22 (91%) and 10/22 (45%) patients received at least four and six cycles of PSMA-RLT, respectively. In these patients (*n* = 20 and *n* = 10), GFR was 55.6 ± 18.8 after four cycles (vs. 44.4 ± 11.0 mL/min at baseline, *p* = 0.007) and 61.8 ± 20.4 mL/min after six cycles (vs. 43.3 ± 9.3 mL/min at baseline, *p* = 0.022), respectively. A representative example is given in Figure 2, showing an increase in GFR after four cycles of PSMA-RLT, concordant with marked reduction of tumor burden and improvement of the patient’s condition.

**Figure 3:**
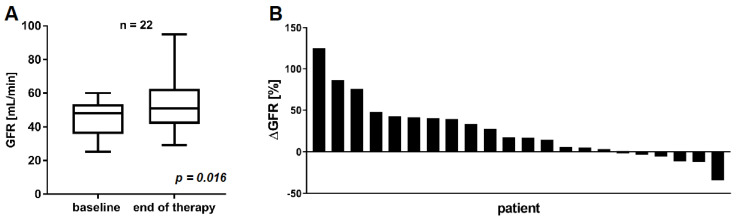
Comparison of GFR at baseline and at the end of [^177^Lu]Lu-PSMA-617 RLT (**A**). Individual change in GFR for each patient from baseline to the end of [^177^Lu]Lu-PSMA-617 RLT (**B**).

After two cycles of PSMA-RLT, 16/22 (72.7%) patients showed partial remission with a PSA decline >50% and 5/22 (22.7%) showed stable disease with a PSA change between −50% and +25%. Only one patient (4.5%) exhibited progressive disease after two cycles with a PSA increase >25%. In the following course, PSMA-RLT was discontinued due to remission in 8/22 (36.4%) patients after a median of four cycles or ended by death in the remaining 14/22 (63.6%) patients due to eventual tumor progression after a median of seven cycles. The GFR at the end of therapy after median of five cycles (range: 3–14, Figure S1) was 54.1 ± 16.7 mL/min and significantly higher compared to baseline GFR (*p* = 0.016) (Figure 3A). A mean GFR increase of +25.3 ± 36.5% was observed. The individual change in GFR of each patient after the last cycle of PSMA-RLT is depicted in Figure 3B. Only one patient (4.5%) revealed a marked GFR decrease (−34.1%) associated with a deterioration of CTCAE grade 2 to grade 3. This patient was of advanced age (88 years), heavily pretreated, and in impaired general condition. The patient started PSMA-RLT with a GFR of 44 mL/min (CTCAE 2), which slowly decreased to 29 mL/min (CTCAE 3) during the course of PSMA-RLT. After an initial biochemical partial response, PSA progression was observed during the course of PSMA-RLT, and the patient died within a short time after the seventh cycle. In all other patients, renal function impairment according to CTCAE grading either decreased (*n* = 10, 45.5%) or remained unchanged (11/22, 50.0%) during and after the end of PSMA-RLT. All CTCAE grades are summed in Table 2.

**Figure 4:**
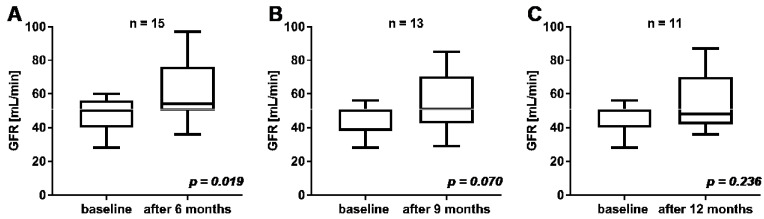
Comparison of GFR at baseline and at follow-up after 6 months (**A**), 9 months (**B**), and 12 months (**C**).

In *n* = 15, 13, and 11 patients the follow-up time after commencement of PSMA-RLT was at least 6, 9, and 12 months, respectively. A statistically significant increase of GFR was observed after 6 months of follow-up compared to mean baseline GFR (57 ± 17.5 mL/min vs. 46.5 ± 10.2 mL/min at baseline, *p* = 0.019; Figure 4). A statistically insignificant increase of GFR was observed after 9 months (55.2 ± 16.8 mL/min vs. 45.2 ± 9.4 mL/min at baseline, *p* = 0.070) and after 12 months (54.6 ± 17.8 mL/min vs. 45.6 ± 8.2 mL/min at baseline, *p* = 0.236).

**Figure 5:**
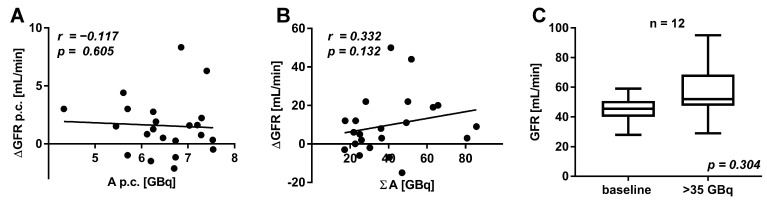
Correlation of the mean administered activity of [^177^Lu]Lu-PSMA-617 per cycle (p.c.) and the change in GFR per cycle (p.c.) (**A**). Correlation of the total administered activity of [^177^Lu]Lu-PSMA-617 and change in GFR (**B**). Comparison of GFR in patients receiving more than 35 GBq [^177^Lu]Lu-PSMA-617 (**C**).

To analyze the dependency of renal function on the applied activity, correlation analyses were performed. No significant correlations between the change in GFR and the mean administered activity per cycle (r = −0.117; *p* = 0.605, Figure 5A) or the total cumulated administered activity (r = 0.332; *p* = 0.132, Figure 5B) were found. Even in patients receiving cumulative activities of more than 35 GBq [^177^Lu]Lu-PSMA-617 (*n* = 12), no significant change of GFR was observed (*p* = 0.304, Figure 5C).

## 4. Discussion

PSMA-RLT is a very effective treatment modality for advanced mCRPC patients with potential nephrotoxicity due to physiological PSMA expression in the renal tubular cells and consecutive radiopharmaceutical binding and retention. Reported absorbed renal doses in PSMA-RLT with ^177^Lu are significant and range between 0.4 and 1.0 Gy/GBq [22,23,24,25,26,27]. However, limited data are available regarding long-term nephrotoxicity of PSMA-RLT, especially in patients with impaired renal function at baseline before initiation of radionuclide therapy. We performed this retrospective analysis on renal function changes in a selected cohort of *n* = 22 mCRPC patients with markedly impaired baseline renal function receiving a median of five RLT cycles with a mean total administered of 40.1 GBq [^177^Lu]Lu-PSMA-617. This is the first PSMA-RLT study regarding the potential nephrotoxicity in a relevant cohort of patients with this grade of compromised baseline renal function and subsequent treatment with high cumulative activities. The analysis reveals that even in these patients the nephrotoxic potential seems to be largely overestimated.

As opposed to prevailing concerns on potential RLT-induced renal toxicity, GFR improved significantly in our cohort of patients with renal impairment (after two, four, and six cycles and at the end of therapy), seemingly due to the overall improvement of patients’ condition. Twelve patients (54.5%) experienced a significant improvement of overall condition during PSMA-RLT documented after two cycles (i.e., when patients were admitted to our therapy ward for the third treatment cycle). A significant improvement of GFR (i.e., change in GFR ≥ 20%) at this time point was statistically associated with clinical improvement (*p* = 0.031, Fisher’s exact test). Additionally, a high PSA response rate (72.7% after two cycles) was noted at this time point in our cohort; however, there was no statistically significant association with the improvement of GFR (*p* = 0.351, Fisher’s exact test); furthermore, there was no correlation between the relative changes in PSA and GFR (r = −0.142, *p* = 0.529). Only in one patient (4.5%), a marked decrease of GFR was noted with a CTCAE-based grade of renal impairment increasing from grade 2 to 3. All other patients showed either unchanged (11/22, 50%) or lower CTCAE (10/22, 45.5%) grades of renal insufficiency at the end of therapy when compared to pre-RLT grades. Additionally, in the follow-up period of 12 months, no significant reduction of GFR was observed. Other studies have shown a similar neglectable negative impact on renal function in patient cohorts with better baseline renal function [28,29]. Zhang et al. observed that [^177^Lu]Lu-PSMA-617 RLT is well tolerated in patients (*n* = 16, 2–6 cycles) with a single functioning kidney without any appearance of CTCAE grade 3 or 4 [28]. Only three patients from this cohort fulfilled the selection criterion of our study, i.e., baseline GFR ≤ 60 mL/min, with no significant reduction seen during follow-up. Yordanova et al. reported no renal impairment or at maximum a slight decrease of GFR from a mixed group of patients (*n* = 55, 3–6 cycles). Only eight patients from this cohort had a reduced baseline GFR as required for inclusion in our study [29]. Additionally, in patients with normal renal function, no relevant nephrotoxicity was reported in various retrospective monocenter studies with a limited number of cycles and patients [11,12,13] and in a larger German multicenter study (*n* = 145) [14]. This is consistent with the results of the Australian prospective phase II trial (initially *n* = 30) by Hofman et al. [15] and its expanded trial report with *n* = 50 patients [16]; their data of adverse effects were limited to a three-month follow-up. From a longer follow-up observation report by Gallyamov et al. with a median of 12 months from start of treatment, the incidence of renal impairment CTCAE grade 1 or 2 is expected to be below 5% [30].

Analogies can also be seen in studies on peptide-receptor-mediated radionuclide therapy (PRRT) in patients with neuroendocrine tumor (NET). There is no acute renal toxicity in patients with metastasized NET [31] when treated with ^177^Lu-labeled somatostatin analogues, including patients with a single functioning kidney [32]. Bodei et al. reported, in a study with *n* = 807 patients, that long term renal impairment was infrequent and especially rare when the radionuclide ^177^Lu was used, as compared to ^90^Y or combinations of both [33].

Our results do not indicate a relationship between the amount of administered per-cycle activity and renal adverse effects as the outcome with regard to renal function did not differ among varying applied treatment activities. This may be seen in line with Seifert et al., who observed no increase of renal side effects whether administering single activities of 6.0 GBq versus 7.5 GBq [^177^Lu]Lu-PSMA-617 per cycle [34]. In our study, even high cumulative activities >35 GBq (up to 85 GBq) were safe and did not lead to further decline of pretreatment-compromised renal function.

These promising results underline that [^177^Lu]Lu-PSMA-617 RLT is associated with a low nephrotoxic potential, even in patients with reduced renal function before initiation of therapy, and even after administration of high cumulative activities in chronic kidney function impairment. However, those patients with chronic renal disease are currently excluded from PSMA-RLT in prospective studies, such as the current phase III VISION trial [35]. In addition, regional treatment guidance, such as the German consensus recommendations [36], advise very restricted use of PSMA-RLT in these patients, which may often lead to preclusion of mCRPC patients from a potentially very successful antitumor treatment. Based on the results of the present retrospective study, we recommend not excluding patients from PSMA-RLT due to renal impairment.

The results reported herein should be considered in the light of some limitations. Our study suffers from the retrospective nature and the small number (*n* = 22) of patients. Another limiting factor is the heterogeneity of the treatment protocol applied to the patients studied, in terms of inequality of therapy dose, number of cycles, and interval between cycles. However, in the absence of published data, this report represents the first relevant renal outcome series of PSMA-RLT in significantly impaired baseline renal function. A further limitation is the lack of analysis of other renal parameters such as cystatin C, which might be superior in calculating GFR or of consecutive renal scintigraphy. Larger studies, ideally in prospective settings, investigating long-term safety of [^177^Lu]Lu-PSMA-617 RLT in patients with impaired renal function, are necessary to address the important issue of how to handle chronic renal failure as an exclusion criterion for this important new treatment modality.

## 5. Conclusions

Nephrotoxic potential of [^177^Lu]Lu-PSMA-617 RLT seems to be largely overestimated, even in mCRPC patients with impaired renal function. In our cohort of mCRPC patients with pre-treatment chronic renal failure or renal function impairment, we observed no RLT-induced negative impact on renal function. Therefore, patients should not be categorically excluded from enrolment to PSMA-RLT due to renal impairment. From our experience, even high cumulative activities of [^177^Lu]Lu-PSMA-617 seem to be well tolerated, with no significant impact on renal function. Ideally, to support the conclusion, all patients with impaired renal function should be treated with a standardized, adapted protocol, which still has to be defined.

## Figures and Tables

**Table 1:** Patient characteristics.

Patient Characteristics	Value	Percentage/Range
**Number of patients**	22	
**Age**		
Median (range) in years	77	(61–88)
≥75 years—*n* (%)	13	(59.1)
**Sites of metastases**—***n* (%)**		
Bone	21	(95.5)
Lymph node	16	(72.7)
Liver	7	(31.8)
**Baseline PSA**		
Median (range) in ng/mL	424	(12–2814)
**Prior treatments**—***n* (%)**		
ADT	22	(100)
Abiraterone	14	(63.6)
Enzalutamide	17	(77.3)
Docetaxel	15	(68.2)
Cabazitaxel	7	(31.8)
**GFR at baseline**—***n* (%)**		
≤60 mL/min	22	(100)
>40 to ≤60 mL/min	15	(68.2)
≤40 mL/min	7	(31.8)

PSA (prostate-specific antigen), GFR (glomerular filtration rate).

**Table 2:** CTCAE grades (version 5.0) for renal function impairment based on GFR at baseline and at the end of PSMA-RLT.

Category	Baseline				End of Therapy			
**Grade**	CTCAE 0°/1°	CTCAE 2°	CTCAE 3°	CTCAE 4°	CTCAE 0°/1°	CTCAE 2°	CTCAE 3°	CTCAE 4°
**GFR** **(mL/min)**	>60	60–30	30–15	<15	>60	60–30	30–15	<15
**Patients**—***n* (%)**	0 (0)	18 (81.8%)	4 (18.2%)	0 (0)	6 (27.3%)	15 (68.2%)	1 (4.5%)	0 (0)

## Data Availability

The datasets used and analyzed during the current study are available from the corresponding author on reasonable request.

## Supplementary Materials

The following is available online https://www.mdpi.com/article/10.3390/cancers13123095/s1, Figure S1: Individual PSMA-RLT course of all patients.
