# Supplementary figures and images for: Renal Safety of [177Lu]Lu-PSMA-617 Radioligand Therapy in Patients with Compromised Baseline Kidney Function
Source: Cancers (Basel). 2021 Jun 21;13(12):3095. doi: 10.3390/cancers13123095 (PMC8235711; doi:10.3390/cancers13123095)

## Supplement

### PSMA-RLT course of all patients

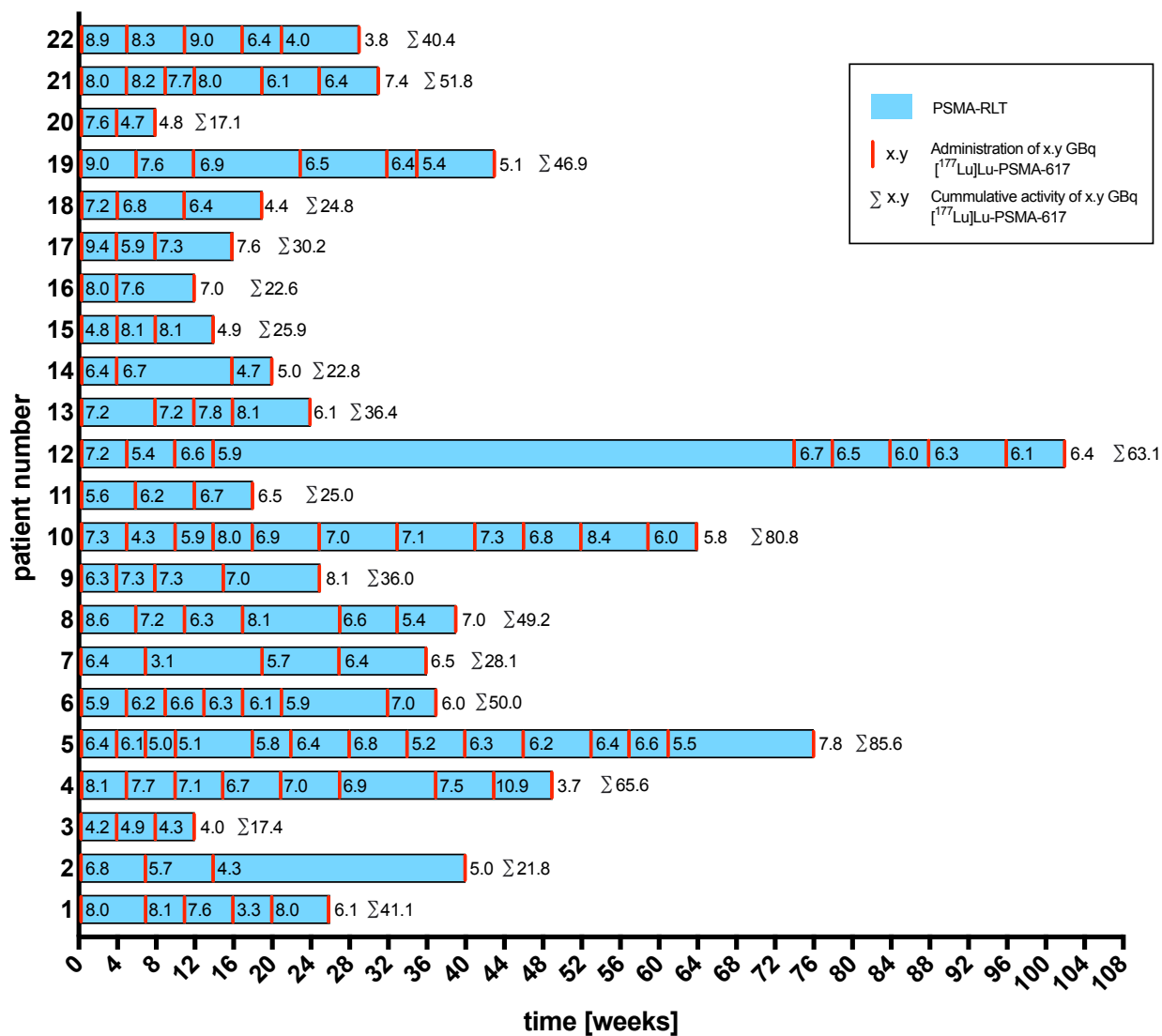

Figure S1: Individual PSMA-RLT course of all patients.

Supplement: Supplementary file 1 [file cancers-13-03095-s001.zip › cancers-1242437-supplementary.pdf]
